# Editorial: Rheumatic fever: 21st century clinical and experimental insights

**DOI:** 10.3389/fcvm.2023.1190372

**Published:** 2023-04-24

**Authors:** Bruno R. Nascimento, Andrea Z. Beaton, Luiza Guilherme, Roney O. Sampaio

**Affiliations:** ^1^ Serviço de Cardiologia e Cirurgia Cardiovascular e Centro de Telessaúde, Hospital das Clínicas da UFMG, Belo Horizonte, MG, Brazil; ^2^ Departamento de Clínica Médica, Faculdade de Medicina da Universidade Federal de Minas Gerais, Belo Horizonte, MG, Brazil; ^3^ Serviço de Hemodinâmica, Hospital Madre Teresa, Belo Horizonte, MG, Brazil; ^4^ Departamento de Cardiopneumologia, Faculdade de Medicina da Universidade de São Paulo (USP), São Paulo, SP, Brazil; ^5^ Instituto do Coração (Incor), Hospital das Clínicas da Faculdade de Medicina da Universidade de São Paulo (HCFMUSP), São Paulo, SP, Brazil; ^6^ The Heart Institute, Cincinnati Children’s Hospital Medical Center, and the University of Cincinnati School of Medicine, Cincinnati, OH, United States

**Keywords:** rheumatic heart disease, epidemiology, pathophisiology, treatment, acute rheumatic fever, immunology and inflammation

**Editorial on the Research Topic** Rheumatic fever: 21st century clinical and experimental insights

## Editorial

The global burden of Rheumatic Heart Disease (RHD) is still high worldwide, and is becoming progressively more concentrated in under-resourced regions (1). As the disease is strictly linked to the socioeconomic background, actions and conditions that ultimately converge to mitigate RHD burden (1) have been more effective in higher-income regions, contributing to its uneven global distribution. Among cardiovascular diseases, RHD accounts for 1.6% of all deaths, resulting in 306,000 fatalities yearly (1), according to updated estimates. Noticeably in the past 2 decades, several research initiatives have been promoted for a deeper understanding not only about RHD, but also about the exaggerated immune event in response to a streptococcal event that precedes RHD, so called acute rheumatic fever (ARF). Thanks to advocacy led by several medical societies and international organizations, there has been growing interest—and consequently funding—for research projects and healthcare programs focused in different fundamental topics, such as: pathophysiology, immune and inflammatory mechanisms, biomarkers, novel diagnostic approaches as echocardiographic (echo) screening and targeted case finding, educational and health promotion interventions, management of early and late phases of disease, novel devices and medications, vaccines and many others.

Beyond allowing for a better understanding of the pathological pathways and processes of ARF and RHD (2), the ongoing programs are globally spreading the importance of RHD and the possibility of its elimination in a lifetime through relatively feasible and simple approaches, bringing together different sectors. Nonetheless, there still remain fundamental gaps in our understanding of ARF and RHD, and some of them were addressed in the special issue of Frontiers in Cardiovascular Medicine: “*Rheumatic Fever: 21st Century clinical and experimental insights*”.

Important data has been presented related to basic science and pathophysiological pathways linked to ARF and RHD. A deep discussion about RHD pathology, immune mechanisms, inflammatory processes at the cell and tissue level, and other underlying mechanisms was provided by Passos et al. in a comprehensive review Passos et al. Evaluating patients pre and post mitral valve commissurotomy due to advanced mitral stenosis (MS), Silva et al. demonstrated that an association between the decrease of specific cytokines and changes in T cell activation with hemodynamic improvement post intervention and long-term outcomes, raising new possibilities for soluble biomarkers of better recovery Silva et al. In this sense, ongoing studies, supported by different funding sources, hold promise in the search for an easily titerable and reproducible biomarker for ARF/RHD, to overcome the need for the combination of clinical observations technological advanced diagnostic tools and non-specific lab tests. As shown by Salie et al. in a meta-analysis of 24 studies, currently available antigens in response to streptococcal infection are not consistently associated with an ARF diagnosis, and further research with more strep proteins is needed Salie et al. Similar observations were reproduced by McGregor et al.: a multi-platform approach to profile circulating autoantibodies denoted marked heterogeneity in autoantibody profiles among ARF patients, although novel candidates were pointed out, in addition to those previously implicated, as myosin and collagens McGregor et al. A translational pilot study by Kirvan et al. involving 23 children (10 with RHD, 6 with Sydenham chorea and 7 with uncomplicated pharyngitis) suggested that group A carbohydrate, N-acetyl-*β*-_D_-glucosamine-specific IgG2 may be an important autoantibody in initial stages of the pathogenesis of streptococcal sequelae, emerging as a future candidate for a biomarker in early disease stages Kirvan et al. Again here, data is still preliminary and derived from limited patient samples. Looking for strategies to overcome these uncertainties, in the perspective article by McMillan et al. the authors propose that with the sharing of multi-region serial blood samples, antibody array technology and T-cell tetramers could lead to the identification of highly specific peptides McMillan et al. However, given the complexity and heterogeneity of tissue involvement by ARF/RHD—a condition restricted to humans—this requires optimal animal models. Rafeek et al. widely discuss the requirements of an ideal animal model—noticeably small rodents—which may potentially mimic the diagnostic criteria features of ARF/RHD, allowing for research on immune responses, biomarker assessment, treatment evaluation and ultimately vaccine development Refeek et al.

On the epidemiological side of research, following the publication of several echocardiographic screening studies from almost all endemic regions of the world in the past 2 decades, doubts still remain about the clinical management of individuals found to have latent RHD in screening. Although the benefits of secondary prophylaxis have been demonstrated by the GOAL trial (3), implementation of such a strategy require further investigations about how to stratify the risk for progression. Adding to the body of evidence about secondary prophylaxis, Torres et al. present a well-characterized cohort of 593 Brazilian children with past ARF, in which 59% evolved with RHD. Regression of mitral and aortic lesions was strongly associated with prophylaxis, and no patients receiving penicillin had progression of valve involvement Torres et al. Also in this issue, Zimmerman et al. present a novel assessment of the risk of latent RHD among schoolchildren with a previous negative screening. Screen-negative individuals (3–5 years prior) had a statistically similar risk of having RHD in a serial echocardiographic screening, although there was a non-significant trend towards a 40% lower risk in this group compared to children with previous positive screening Zimmerman et al. However, the decision whether serial screening should be recommended should consider these data, but also warrants further investigation.

Adding to the knowledge about the incidence of ARF in non-endemic countries, the study by Marino et al. in Monza, Italy, depict a 10-year incidence of ARF in a retrospective analysis of 70 reported cases. The mean rate in schoolchildren between 5 and 14 years was 5.7/100,000, considerably above the threshold proposed by the World Heart Federation for low-risk areas Marino et al., and reinforcing the need for constant monitoring of disease burden even in places where it near eradicated. On the other hand, the data by Opara et al. depict the economic burden posed by RHD in under sourced areas as Uganda, with overall direct and indirect costs of around USD 78 per patient/year, markedly affecting the poorer areas, where the utilization of financial coping mechanisms is frequent Opara et al.

In terms of imaging, much has been learned from studies that have been applying multi-modality methods and technology-based add-ons and tools, previously validated in other structural heart diseases, for patients with RHD. Beyond diagnostic refinement and planning of interventional procedures, advanced imaging has been contributing for the understanding of the underlying pathological mechanisms of the disease. As an example of this matter, Rosa et al. show preliminary data from with 25 patients suggesting that myocarditis due to ARF reactivation may be a cause of echo-detected left ventricular dysfunction regardless of the degree of valvular involvement Rosa et al. Given the possible reversibility with corticosteroid treatment, the acknowledgement of this relationship is crucial, and serial examinations should be considered in this patient subset. Expanding the possibilities of cardiac imaging, tridimensional (3D) echocardiography is also being progressively more applied for RHD management. As pointed out in a comprehensive review paper by Vieira et al., up-to-date 3D echocardiography is capable to provide additional anatomical and morphofunctional information about patterns of rheumatic valvular involvement, being relevant not only for diagnostic purposes but also for prognostication and guidance of invasive procedures—as mitral commissurotomy—and correction of peri-procedural complications Vieira et al. The authors postulate that, with adequate equipment and training, 3D echo is a ready-to-use technique for rheumatic patients with valvular abnormalities. Indeed, 3D imaging has been enabling for significant advances as a guidance for interventional cardiology procedures. Although the pathological process of RHD differs widely from that of non-rheumatic degenerative valve disease, especially in terms of calcification, involvement of the sub valvular apparatus and adjacent structures, and especially the younger age of patients, percutaneous valve replacement has been more commonly indicated in selected cases. Besides case reports and series showing good results of transcatheter aortic valve replacement (TAVR) in rheumatic patients (4), valve-in-valve procedures are being tested in bioprosthetic valve dysfunction. In the case series presented by Lopes et al., including interventions in mitral, aortic and tricuspid positions, RHD patients had similar procedural success compared to non-rheumatic individuals, but 30-day mortality rates were higher. At 20 months, however, cumulative mortality rates were superimposable Lopes et al., suggesting that this may be an option to reduce the morbidity of redo procedures in young patients living with RHD, but randomized data are required.

Finally, the broad and growing scope of research initiatives related to RHD worldwide, notably boosted by implementation science and international collaboration have resulted in outstanding magnification of accords, statements and resolutions that contribute to pave the road to eradicate the disease in a lifetime. As an example, the “2017 Cairo Accord” exemplary defined policy priorities for fighting ARF/RHD and built on a recent series of broad initiatives and calls to action, as detailed in the review paper by Kotit et al. Along with a series of other fundamental statements, the accord culminated in the recognition of ARF/RHD as global health priorities in the global stage following the 2018 World Heart Assembly. Including a broad span of ongoing research on this topic, from basic science to clinical and population studies, this special issue of Frontiers in Cardiovascular Medicine (a summary of the published articles is presented in Table 1) is aimed at contributing to the superb scientific moment faced by ARF/RHD, also as a call-to-action for continuing collaborative efforts needed for the mitigation of this complex disease of the poor.

**Table 1:** Manuscripts published in the special issue: rheumatic fever: 21st century clinical and experimental insights.

Pathophysiology/Immunology:	Reference:
Review on pathophysiology and immunology	Passos et al.
Cytokines and changes in T cell activation after intervention	Silva et al.
Antigens in response to Streptococcal infection (metanalysis)	Salie et al.
Heterogeneity in autoantibody profile in ARF	McGregor et al.
N-acetyl-*β*-_D_-glucosamine-specific IgG2 autoantibody in early stages of ARF	Kirvan et al.
Identification of specific peptides using multiregion blood samples	McMillan et al.
Animal model for ARF	Refeek et al.
**Epidemiology**	
Regression of valve disease after Penicillin prophylaxis	Torres et al.
Risk of latent RHD in schoolchildren with previously negative screening	Zimmerman et al.
Incidence of ARF in Northern Italy	Marino et al.
Costs of RHD in Uganda	Opara et al.
The “Cairo Accord” and other global health initiatives for reducing RHD burden	Kotit et al.
**Echocardiography/Treatment**	
Myocarditis during ARF reactivation	Rosa et al.
3D echocardiography for RHD	Vieira et al.
Valve-in-valve interventions in patients with RHD	Lopes et al.

ARF, acute rheumatic fevere; RHD, rheumatic heart disease.

## References

[1] Roth GA Mensah GA Johnson CO Addolorato G Ammirati E Baddour LM Global burden of cardiovascular diseases and risk factors, 1990–2019: update from the GBD 2019 study. J Am Coll Cardiol. (2020) 76:2982–3021. 10.1016/j.jacc.2020.11.010 33309175 PMC7755038

[2] Rwebembera J Nascimento BR Minja NW de Loizaga S Aliku T Dos Santos LPA Recent advances in the rheumatic fever and rheumatic heart disease Continuum. Pathogens. (2022) 11(2):179. 10.3390/pathogens11020179 35215123 PMC8878614

[3] Beaton A Okello E Rwebembera J Grobler A Engelman D Alepere J Secondary antibiotic prophylaxis for latent rheumatic heart disease. N Engl J Med. (2022) 386:230–40. 10.1056/NEJMoa2102074 34767321

[4] Mentias A Saad M Desai MY Krishnaswamy A Menon V Horwitz PA Transcatheter versus surgical aortic valve replacement in patients with rheumatic aortic stenosis. J Am Coll Cardiol. (2021) 77:1703–13. 10.1016/j.jacc.2021.02.032 33832596 PMC8043573

